# Missed injury and complication after laparoscopy in trauma: Is the procedure still preferable?

**DOI:** 10.4103/0972-9941.72603

**Published:** 2010

**Authors:** Viroj Wiwanitkit

**Affiliations:** Department of Laboratory Medicine, Faculty of Medicine, Chulalongkorn University, Bangkok 10330, Thailand

Dear Sir,

Use of laparoscopy in trauma is still a controversial issue. The high rate of missed injury and possibility of induction of severe complications should be carefully considered. In the recent publication by Umman, the unfavourable outcome can also be seen.[1] The suggestion that “the best applications may be in haemodynamically stable patients with stab wounds or tangential wounds to the anterior abdominal wall”[1,2] should be revised. Although the complication due to the procedure might not be highly prevalent and might relate to the specific difficulty in specific case and experience of the practitioner, the very high rate of missed injury might be a very big problem. Based on the data that “approximately 5% of patients with blunt trauma and 10% to 15% of patients with penetrating injuries to the chest and abdomen have diaphragmatic injuries”[1,3] and “as a diagnostic tool, laparoscopy had a 41% to 77% missed injury rate per patient,”[1,4] it can be estimated that missed diaphragmatic injuries can be seen in 2.1% to 3.9% of patients with blunt trauma and 4.1% to 11.6% of patients with penetrating injuries to the chest and abdomen if diagnostic laparoscopy is used. For sure, the high rate of missed injury means repeated abdominal exploration later on, and medical litigation might be another issue that should be kept in mind. Hence laparoscopy as minimal access surgery should have limited role and usefulness in trauma.
